# Autophagy-Related Signatures as Prognostic Indicators for Hepatocellular Carcinoma

**DOI:** 10.3389/fonc.2022.654449

**Published:** 2022-03-24

**Authors:** Wen Ye, Zhehao Shi, Yilin Zhou, Zhongjing Zhang, Yi Zhou, Bicheng Chen, Qiyu Zhang

**Affiliations:** ^1^ Department of Breast Surgery, The Second Affiliated Hospital and Yuying Children’s Hospital of Wenzhou Medical University, Wenzhou, China; ^2^ Department of Hepatobiliary Surgery, The First Affiliated Hospital, Wenzhou Medical University, Wenzhou, China; ^3^ College of Engineering, Boston University, Boston, MA, United States; ^4^ Key Laboratory of Diagnosis and Treatment of Severe Hepato-Pancreatic Diseases of Zhejiang Province, The First Affiliated Hospital, Wenzhou Medical University, Wenzhou, China

**Keywords:** HCC (hepatic cellular carcinoma), autophagy, prognostic signature, FKBP1A, TCGA, ICGC

## Abstract

**Background:**

Hepatocellular carcinoma (HCC) is the most common and deadly type of liver cancer. Autophagy is the process of transporting damaged or aging cellular components into lysosomes for digestion and degradation. Accumulating evidence implies that autophagy is a key factor in tumor progression. The aim of this study was to determine a panel of novel autophagy-related prognostic markers for liver cancer.

**Methods:**

We conducted a comprehensive analysis of autophagy-related gene (ARG) expression profiles and corresponding clinical information based on The Cancer Genome Atlas (TCGA) and International Cancer Genome Consortium (ICGC) databases. The univariate Cox proportional regression model was used to screen candidate autophagy-related prognostic genes. In addition, a multivariate Cox proportional regression model was used to identify five key prognostic autophagy-related genes (ATIC, BAX, BIRC5, CAPNS1, and FKBP1A), which were used to construct a prognostic signature. Real-time qPCR analysis was used to evaluate the expression levels of ARGs in 20 surgically resected HCC samples and matched tumor-adjacent normal tissue samples. In addition, the effect of FKBP1A on autophagy and tumor progression was determined by performing *in vitro* and *in vivo* experiments.

**Results:**

Based on the prognostic signature, patients with liver cancer were significantly divided into high-risk and low-risk groups in terms of overall survival (OS). A subsequent multivariate Cox regression analysis indicated that the prognostic signature remained an independent prognostic factor for OS. The prognostic signature possessing a better area under the curve (AUC) displayed better performance in predicting the survival of patients with HCC than other clinical parameters. Furthermore, FKBP1A was overexpressed in HCC tissues, and knockdown of FKBP1A impaired cell proliferation, migration, and invasion through the PI3K/AKT/mTOR signaling pathway.

**Conclusion:**

This study provides a prospective biomarker for monitoring outcomes of patients with HCC.

## Background

Liver cancer is one of the most common malignant tumors in the alimentary (digestive) system, has a poor clinical prognosis, and is the second leading cause of cancer-related mortality. Hepatocellular carcinoma (HCC) accounts for 75%–85% of primary liver cancer cases, making it the most common type of liver cancer (1). Patients are often diagnosed with liver cancer at a late stage, and thus the efficacy or result of the operation is limited and poor. Thus, an effective therapeutic indicator must be explored to achieve a better prognosis in the clinic. The correlation between autophagy and HCC has been studied, providing a new direction and approach for the clinical treatment of liver cancer. For example, autophagy has been shown to induce the resistance of HCC cells to chemotherapeutic agents (2, 3).

Autophagy is the process of transporting damaged, denatured, or aging cellular components into lysosomes for digestion and degradation. Autophagy is an approach or process regulating cell metabolism that maintains homeostasis and participates in a variety of pathophysiological processes, including malignant tumors. However, the efficacy of autophagy in cancer has not yet been conclusively determined, and autophagy has been reported to have a dual function in the development of tumors. On the one hand, autophagic degradation of cellular components may provide a nutrient supply for tumor cell survival. On the other hand, it inhibits tumorigenesis by clearing toxic cellular materials. To date, researchers investigating autophagy and liver cancer have mainly focused on cancer progression and chemotherapy resistance (4–6). However, the role of autophagy in the liver cancer prognosis has rarely been explored.

In this study, we established a signature involving five autophagy-related genes (ARGs) to predict the prognosis of patients with liver cancer and found that it was an independent prognostic index for patients with liver cancer. Our study suggests that ARGs play important roles in liver cancer and may show potential and valuable performance for predicting the prognosis of patients with HCC.

## Materials and Methods

### Data Acquisition

Two hundred thirty-two ARGs were obtained from the Human Autophagy Database (HADb, http://autophagy.lu/clustering/index.html). The transcription factor (TF) information, gene expression profiles, and clinical data of the liver cancer cohort were obtained from The Cancer Genome Atlas (TCGA) (https://portal.gdc.cancer.gov/) and International Cancer Genome Consortium (ICGC) portals (http://dcc.icgc.org/releases/current/Projects), respectively. At the same time, the samples acquired from TCGA dataset were used as the training group, and those from the ICGC dataset were used as the validation cohort.

### Identification of Differentially Expressed ARGs

An analysis of differentially expressed ARGs between HCC and normal liver tissue was conducted using the “limma” package of R software. Genes with false discovery rate (FDR) < 0.05 and |log2 fold change (FC)| > 1 were considered significantly differentially expressed ARGs. Then, a Venn diagram was constructed to detect overlapping target genes.

### Enrichment Analysis of Differentially Expressed ARGs

The Kyoto Encyclopedia of Genes and Genomes (KEGG) pathway genome and Gene Ontology (GO) genome were selected to conduct a series of gene functional enrichment analyses to identify the major biological attributes. In addition, we employed the GOplot package to visualize the enriched terms.

### Construction of an OS Risk Prognostic Model Based on ARGs

Initially, the potential prognostic ARGs related to the OS of patients with liver cancer were selected by performing univariate Cox regression analyses. Next, multivariate Cox regression analyses were used to explore whether these candidate prognostic genes were independent predictors of the prognosis in TCGA cohorts. Finally, several prognostic ARGs were selected (included or excluded), and the Cox proportional hazard regression model was employed to build the OS risk prognostic model. The autophagy-related risk score for each patient was calculated by multiplying the relative expression level by the regression coefficients. X-tile analysis was used to select the optimal cutoff value for the training group. Simultaneously, patients with HCC were separated into low-risk and high-risk groups according to the value. Overall survival was assessed using Kaplan–Meier curves. Then, the differences in survival were estimated into high- or low-risk groups based on the log-rank test. The Cox regression model was employed to analyze the factors affecting the OS of patients with HCC. The risk score, age, sex, cancer grade, and cancer stage were used as covariates.

### Human Tissues

Twenty paired liver cancer and matched normal adjacent tissue samples were obtained from patients who underwent surgical resection at the First Affiliated Hospital of Wenzhou Medical University (Wenzhou, China). The clinicopathological features of all samples analyzed in this study were confirmed as hepatocellular carcinoma. All specimens were frozen in liquid nitrogen. Ethical approval was confirmed by the ethical committee of the hospital, and written informed consent was obtained from each patient.

### Quantitative Real-Time PCR

Total RNA was isolated from tissues using TRIzol reagent (Invitrogen, Carlsbad, CA, USA). Reverse transcription was performed using the High-Capacity cDNA Reverse Transcription Kit (RR036A, Takara Bio, San Jose, CA, USA). PCR was performed with a 7500 Fast Real-Time PCR System (Applied Biosystems, Foster City, CA, USA) using SYBR™ Green PCR Master Mix (RR036A, TAKARA Bio, USA). **Table S3** lists the primers used in this study.

### Cell Lines and Culture

The human hepatic stellate cell line LX2, normal liver cell line L02, and HCC cell lines MHCC97L, MHCC97H, HCCLM3, Hep3B, PLC/PRF/5, and Huh7 were obtained from the Academy of Sciences Cell Bank of China. The cells were cultured in RPMI 1640 or DMEM supplemented with 10% FBS and 1% penicillin/streptomycin at 37°C with 5% CO_2_.

### Cell Transfection

Cells were transfected with FKBP1A knockdown plasmids using Lipofectamine 3000 (L3000008; Invitrogen) according to the manufacturer’s protocol. Cells in logarithmic growth were seeded in 6-well plates, and transfection was performed when the cells reached 70%–80% confluence. After an incubation at 37°C for 48 h, FKBP1A expression was evaluated.

### Cell Counting Kit-8 Assay

Forty-eight hours after transfection, every group of MHCC97H cells was inoculated into a 96-well plate at a density of 5,000 cells/well. Five replicates were established for each group. A Cell Counting Kit-8 (CCK-8) cell proliferation and cytotoxicity test kit (CK04, Dojindo, Kumamoto, Japan) was used, and absorbance values were also detected after 0, 24, 48, and 72 h of culture.

### Colony-Formation Assay

Transfected MHCC97H cells in the logarithmic growth phase were seeded in six-well plates at a density of 500–1,000 cells per well. When individual cells grew into colonies that were visible by eye, cells were stained with 0.1% crystal violet (Meilunbio, Dalian, China). The number of clones was counted using an inverted microscope.

### Transwell Assays

The migratory and invasive capacities of cells were determined using transwell assays. Forty-eight hours after transfection, MHCC97H cells (2 × 10^4^) were plated in the upper chambers of an 8-μm pore polycarbonate membrane (Corning Costar Corp, Corning, NY, USA) coated with or without 50 μl of Matrigel (BD Biosciences, San Jose, CA, USA). DMEM containing 10% FBS was added to the lower chamber as a chemoattractant. After an incubation for 24 h for migration and 48 h for invasion at 37°C, the cells on the upper surface of the filter were gently removed, and cells that migrated to the bottom of the membrane were fixed with 4% paraformaldehyde, stained with a crystal violet solution, and counted under a microscope at ×200 magnification. The numbers of cells counted in five random fields were averaged.

### Wound Healing Assay

HCC cells were seeded in six-well plates and cultured in medium containing 1% fetal bovine serum. The cell monolayer was scratched with a 100-µl pipette tip. Wound healing was then observed and photographed at 0, 24, 48, and 72 h after injury using a microscope (TS100-F, Nikon, Tokyo, Japan).

### Animal Xenograft Model

BALB/C nude mice (18–20 g) were purchased from Wenzhou Medical University, and animal experiments conformed to the Institutional Ethical Guidelines for Animal Experiments. Xenografting was performed by subcutaneous implantation of MHCC97H cells (1 × 10^6^/0.1 ml) transfected with sh-FKBP1A or sh-NC into the right flanks of the mice. After 14 days, tumor growth was examined every 2 days, and tumor volume was calculated using the formula V = 1/2 (width2× length). Finally, the mice were euthanized, and the weight of the tumor tissues was measured.

### Western Blot Analysis

Total protein was lysed in RIPA buffer (Beyotime, Shanghai, China) in the presence of PMSF (Beyotime) and PhosSTOP (Roche, Basel, Switzerland). Western blots were carried out according to standard procedures. Antibodies against ATG5, ATG7, and Beclin-1 were obtained from Abcam (Cambridge, UK). Antibodies against SQSTM1/p62 were obtained from Proteintech (Rosemont, IL 60018, USA). Antibodies against LC3B, PI3K, p-PI3K, AKT, p-AKT, mTOR, p-mTOR, and GAPDH were obtained from Cell Signaling Technology (CST, Danvers, MA, USA).

### Immunohistochemical Staining

Paraffin-embedded sections of mouse tumor tissue were subjected to immunohistochemical staining (IHC) following standard protocols. Antibodies against Ki67 (Abcam) and LC3B (CST) were used. Representative images were captured by a Leica DM4000B microscope (Jena, Germany).

### Statistical Analysis

The cutoff point for the risk score was determined using X-tile 3.6.1 software based on the minimal p value. Statistical analyses were performed with R software. A receiver operating characteristic (ROC) curve was constructed to estimate the prognostic value of the risk score. Area under the curve (AUC) values equal to or greater than 0.7 were regarded as a significant predictive value.

Experimental data are presented as the means ± standard deviations (SD). All statistical analyses were performed using GraphPad 8 software. Two-sided Student’s t test and ANOVA were used for statistical analyses. A p value less than 0.05 was considered statistically significant.

## Results

### Identification of Differentially Expressed ARGs

**Figure 1:**
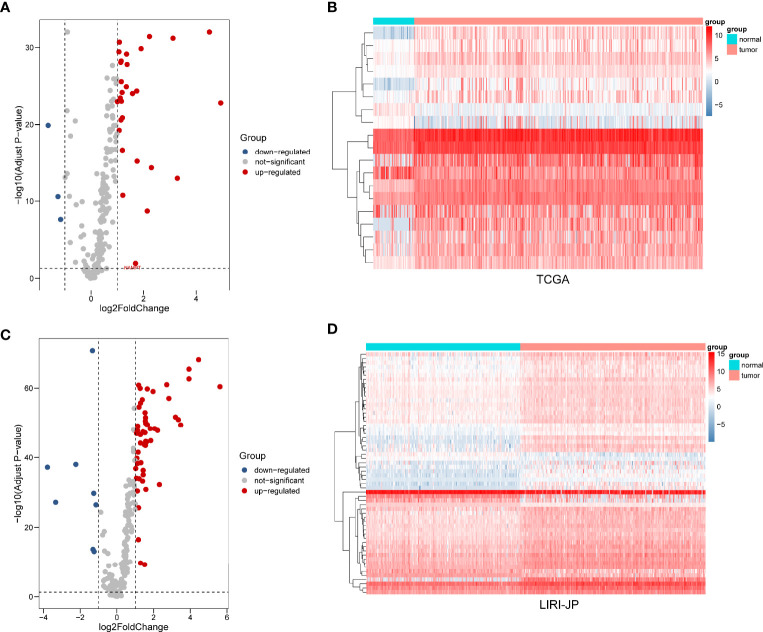
Differential expression of autophagy-related genes (ARGs) between liver cancer and normal liver tissues in two databases. **(A)** The volcano plot and **(B)** clustering analysis of differentially expressed ARGs for TCGA database. **(C)** The volcano plot and **(D)** clustering analysis of differentially expressed ARGs for the ICGC database. Each point represents a gene. The red dots represent significantly upregulated ARGs, and the blue dots represent significantly downregulated ARGs. Each column represents one sample, and each row represents one gene.

**Figure 2:**
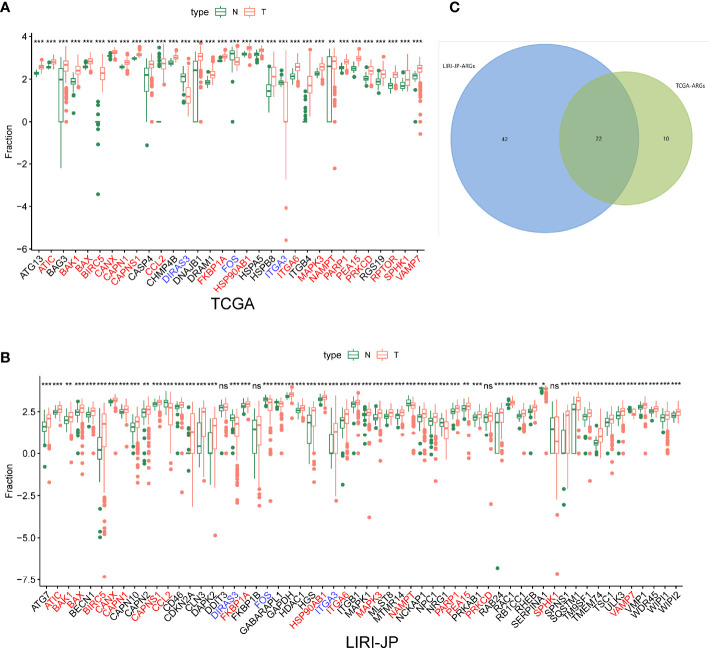
The expression patterns of differentially expressed autophagy-related genes (ARGs) in liver cancer and normal liver tissues from two databases. **(A)** The expression patterns in TCGA dataset. **(B)** The expression patterns in the ICGC dataset. **(C)** Venn diagrams of the overlapping differentially expressed ARGs between TCGA and ICGC databases, including 3 significantly upregulated genes and 19 significantly downregulated genes. Ns, not significant, *p < 0.05, **p < 0.01, ***p < 0.001.

After extracting the expression levels of 232 ARGs from patients with liver cancer, 32 and 64 differentially expressed ARGs were identified in TCGA and ICGC databases, respectively (**Figure 1**). Furthermore, the expression pattern of differentially expressed ARGs between HCC tissues and normal tissues in these two databases was visualized using scatter plots (**Figures 2A, B**). A Venn diagram was constructed to identify the differentially expressed ARGs in both TCGA and ICGC databases (**Figure 2C**). Finally, we revealed 22 common differentially expressed ARGs in the two datasets, consisting of 3 upregulated genes (DIRAS3, FOS, and ITGA3) and 19 downregulated genes (ATIC, BAK1, BAX, BIRC5, CANX, CAPN1, CAPNS1, CCL2, FKBP1A, HSP90AB1, ITGA6, MAPK3, NAMPT, PARP1, PEA15, PRKCD, RPTOR, SPHK1, and VAMP7).

**Figure 3:**
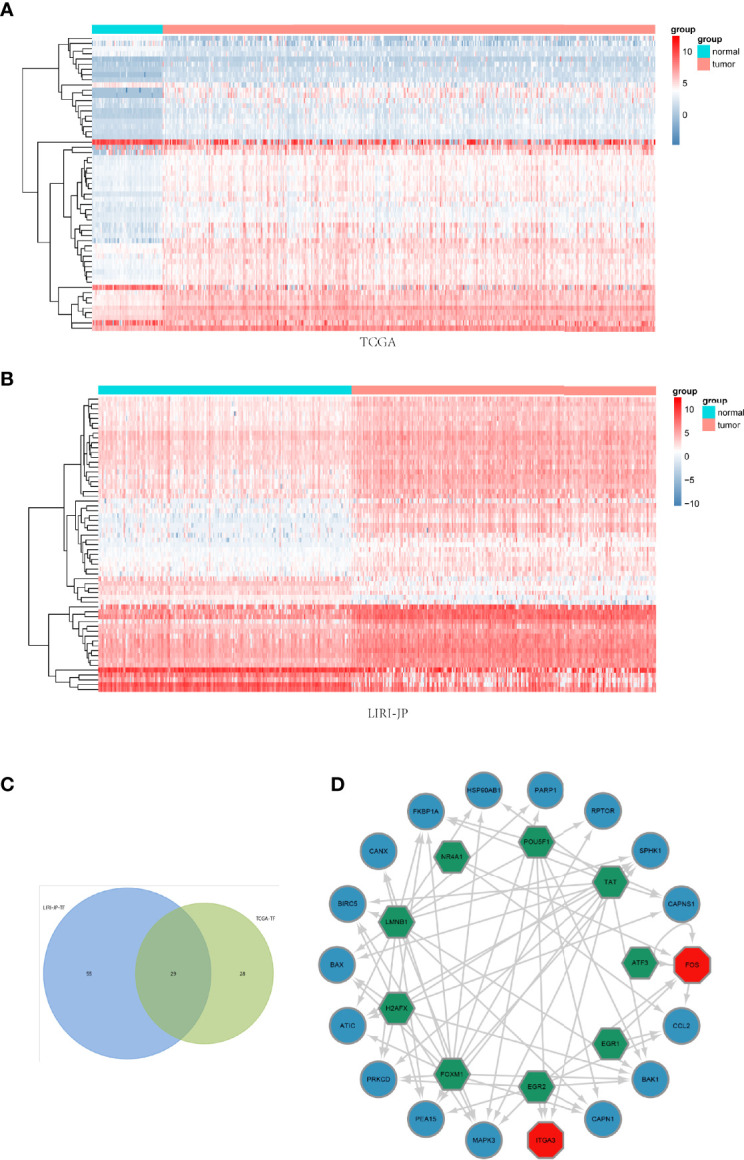
Coexpression network of the differentially expressed transcription factor (TF) genes and differentially expressed autophagy-related genes (ARGs). **(A, B)** Clustering analysis of differentially expressed TFs for TCGA and ICGC databases. **(C)** Venn diagrams of the overlapping differentially expressed TFs between TCGA and ICGC datasets. **(D)** The coexpression networks. Green hexagons represent TF genes, blue circles represent low-risk ARGs, and red octagons represent high-risk ARGs.

After differentially expressed TF genes were screened in TCGA and ICGC databases, a Venn diagram was generated to identify the intersecting genes (**Figures 3A–C**). Then, a coexpression network was established with these differentially expressed TF genes and ARGs (**Figure 3D**). The results showed that differentially expressed ARGs were coexpressed interactively with ERG1, ERG2, and FOXM1.

### Functional Enrichment Evaluation of the Differentially Expressed ARGs

**Figure 4:**
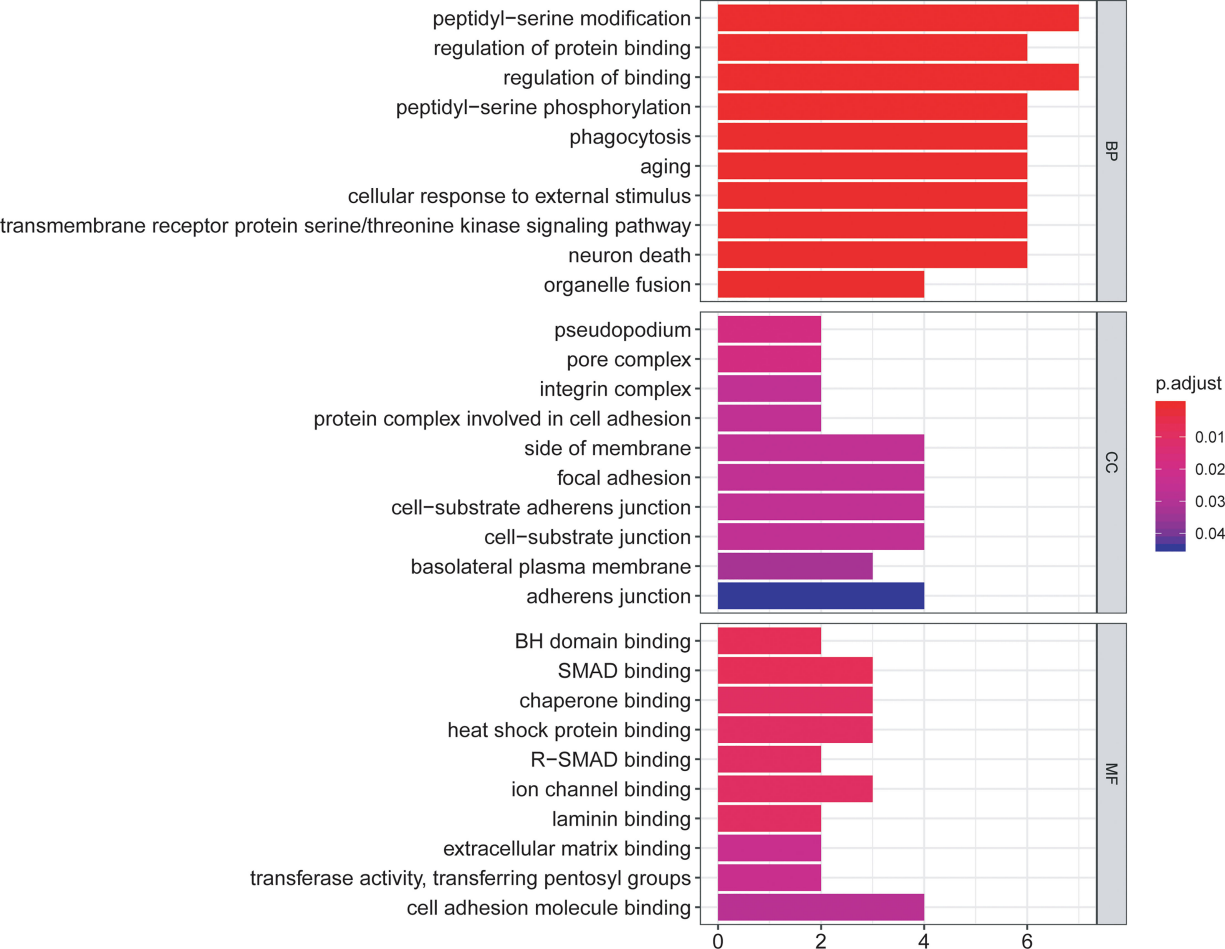
The bar plot of enriched Gene Ontology (GO) terms. BP indicates biological process, CC indicates cellular component, and MF indicates molecular function.

**Figure 5:**
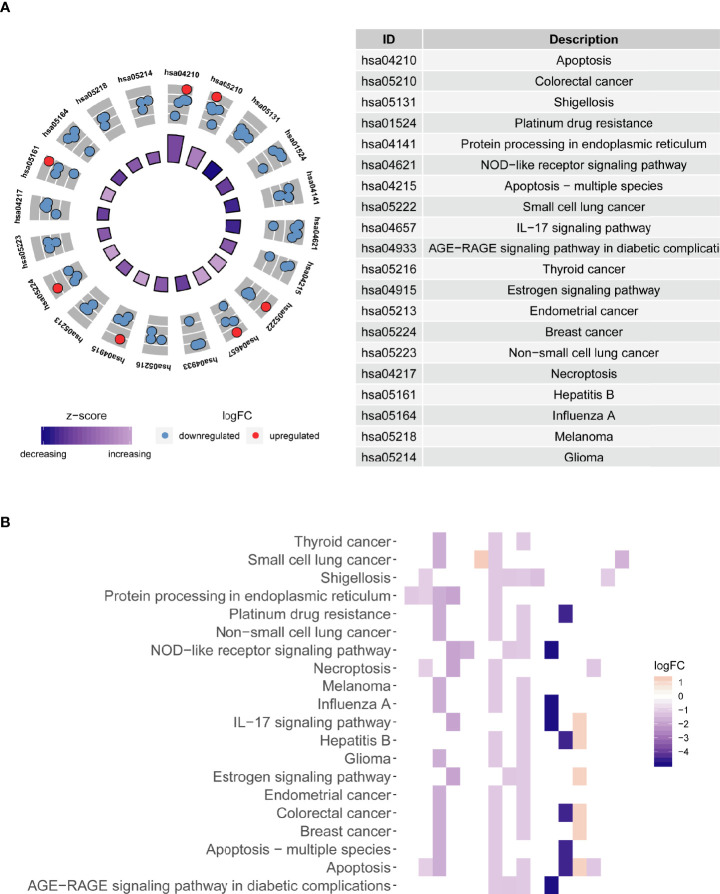
Kyoto Encyclopedia of Genes and Genomes (KEGG) pathway enrichment analysis of differentially expressed autophagy-related genes (ARGs). **(A)** The circle shows a scatter plot for each term of the logFC of the assigned genes. The red circles represent upregulation, and the blue circles represent downregulation. **(B)** Heatmap of the relationship between ARGs and pathways. The color of each block depends upon the values of logFC.

The functional enrichment analysis of those differentially expressed ARGs was performed using GO and KEGG pathway analyses, which elucidated the biological functions (**Figures 4**, **5**). The top enriched GO annotations were related to peptidyl-serine modification, regulation of protein binding, and regulation of binding during the biological process. Cellular components included pseudopodia, pore complexes, and integrin complexes. The terms BH domain binding, SMAD binding, and chaperone binding were the top three molecular functions identified. The enriched KEGG pathways were notably associated with apoptosis, colorectal cancer, Shigellosis, platinum drug resistance, protein processing in the endoplasmic reticulum, and other pathways. Most of the Z scores of the enriched pathways were less than zero, suggesting that most of them would be decreased. For differentially expressed ARGs, the KEGG pathways including the IL-17 signaling pathway and the AGE−RAGE signaling pathway are displayed in a heatmap.

### Identification of Prognostic ARGs

**Figure 6:**
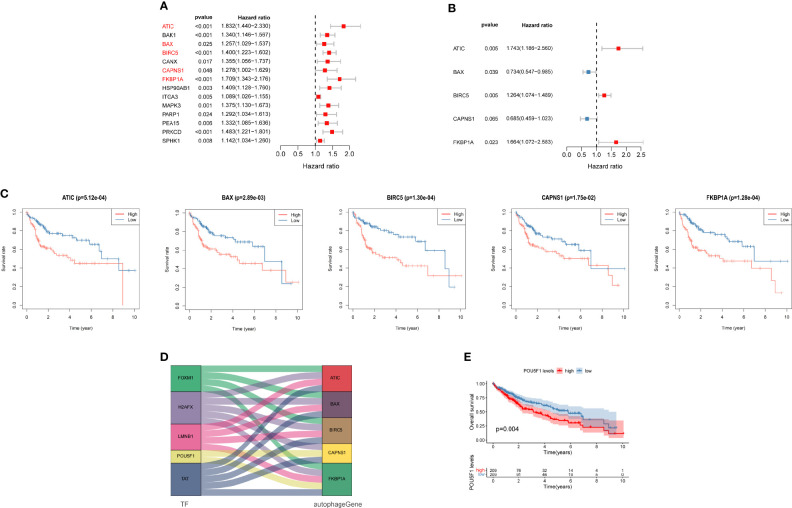
Autophagy-related prognostic genes for patients with liver cancer. **(A)** Univariate and **(B)** multivariate Cox regression analyses revealed the pool of prognosis-related genes. **(C)** Analysis of the Kaplan–Meier survival curve for TCGA cohort. **(D)** Mulberry map of transcription factors and autophagy-related genes (ARGs). **(E)** Analysis of the Kaplan–Meier survival curve for POU5F1.

We evaluated the data obtained from TCGA using a univariate Cox regression analysis to reveal the relationship between the prognosis of patients and differential ARG expression profiles. Fourteen ARGs were concurrently significantly associated with the prognosis of patients with liver cancer (**Figure 6A**). Then, a multivariate Cox regression analysis was subsequently performed (**Figure 6B**). Finally, five genes, ATIC, BIRC5, BAX, CAPNS1, and FKBP1A, were identified as risk genes for OS and used to develop a prognostic signature. Moreover, Kaplan–Meier analysis was performed to focus on the prognostic ability of each ARG. The results from TCGA database indicated that overexpression of ATIC, BIRC5, BAX, CAPNS1, and FKBP1A was closely related to an inferior OS of patients with liver cancer (**Figure 6C**). The results of the mulberry map showed that the FOXM1, H2AFX, LMNB1, POU5F1, and TAT transcription factors were related to the five genes (**Figure 6D**). In addition, they all regulated the expression of FKBP1A. We subsequently conducted a prognostic analysis. The results showed that high expression of the transcription factor POU5F1 indicated a poor prognosis (**Figure 6E**).

### Establishment and Definition of the Prognostic Risk Model

**Figure 7:**
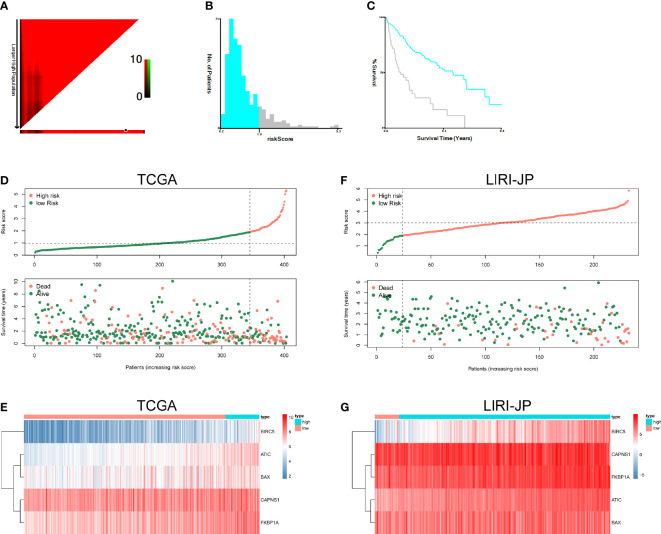
Autophagy-related risk score analysis of patients with HCC. X-tile analysis of the prognostic risk score in TCGA training cohort. **(A)** The X‐tile plots of training group. The black circles highlight the optimal cutoff values. **(B)** Histogram of the entire cohort divided into low-risk score and high-risk score subgroups according to the cutoff value of 1.9. Blue bars represent the low-risk score group, and gray bars represent the high-risk score group. **(C)** Kaplan–Meier plot of overall survival (OS) in groups stratified using the optimal cutoff value of the risk score. Blue curves represent the low-risk score group, and gray curves represent the high-risk score group. **(D)** The risk score distribution and OS of patients in TCGA database. **(E)** Heatmap of the expression profiles of the five genes in TCGA database. **(F)** The risk score distribution and OS of patients in the ICGC database. **(G)** Heatmap showing the expression profiles of the five genes in the ICGC database.

The risk score formulas for OS-based prognosis were constructed as follows: risk score = (0.5554 × ATIC expression level) + (-0.3096 × BAX expression level) + (0.2345 × BIRC5 expression level) + (-0.3780 × CAPNS1 expression level) + (0.5091 × FKBP1A expression level). Subsequently, X-tile analysis was performed and identified 1.9 as the optimal cutoff point for the risk score in TCGA set (**Figures 7A–C**). Using the cutoff point calculated from the risk score of each patient in TCGA cohorts, we categorized them into two groups: a high-risk group and a low-risk group. The risk score distribution and survival status of patients in TCGA dataset are shown in **Figure 7D**. Next, the heatmaps showed the expression profiles of the five risk ARGs (**Figure 7E**). We observed that high-risk patients exhibited higher expression levels of risk genes (ATIC, BIRC5, BAX, CAPNS1, and FKBP1A). The results obtained from the ICGC dataset were similar (**Figures 7F, G**).

The correlations between the risk model and clinical parameters in patients with HCC are summarized in **Table 1**. The histological grade (p = 0.029), pathological T stage (p < 0.001), and pathological stage (p < 0.001) were closely correlated with the risk score.

**Table 1:** Clinical characteristics of liver cancer patients in The Cancer Genome Atlas set.

Characteristics	High-risk (n = 30)	Low-risk (n = 205)	p value
Age			0.535
>65 (n, %)	22 (73.3%)	134 (65.4%)	
≤65 (n, %)	8 (26.7%)	71 (34.6%)	
Gender			0.532
Male (n, %)	11 (36.7%)	63 (30.7%)	
Female (n, %)	19 (63.3%)	142 (69.3%)	
Histological grade			0.029
G1 + G2 (n, %)	11 (36.7%)	121 (59.0%)	
G3 + G4 (n, %)	19 (63.3%)	84 (41.0%)	
Pathological stage			<0.0001
I + II (n, %)	11 (36.7%)	152 (74.1%)	
III + IV (n, %)	19 (63.3%)	53 (25.9%)	
T stage			<0.0001
T1 + T2 (n, %)	12 (40.0%)	155 (75.6%)	
T3 + T4 (n, %)	18 (60.0%)	50 (24.4%)	
N stage			1.000
N0 (n, %)	30 (100.0%)	201 (98.0%)	
N1 (n, %)	0 (0.0%)	4 (2.0%)	
M stage			0.423
M0 (n, %)	29 (96.7%)	202 (98.5%)	
M1 (n, %)	1 (3.3%)	3 (1.5%)	

### The Prognostic Risk Model Is an Independent Predictor of Liver Cancer

**Figure 8:**
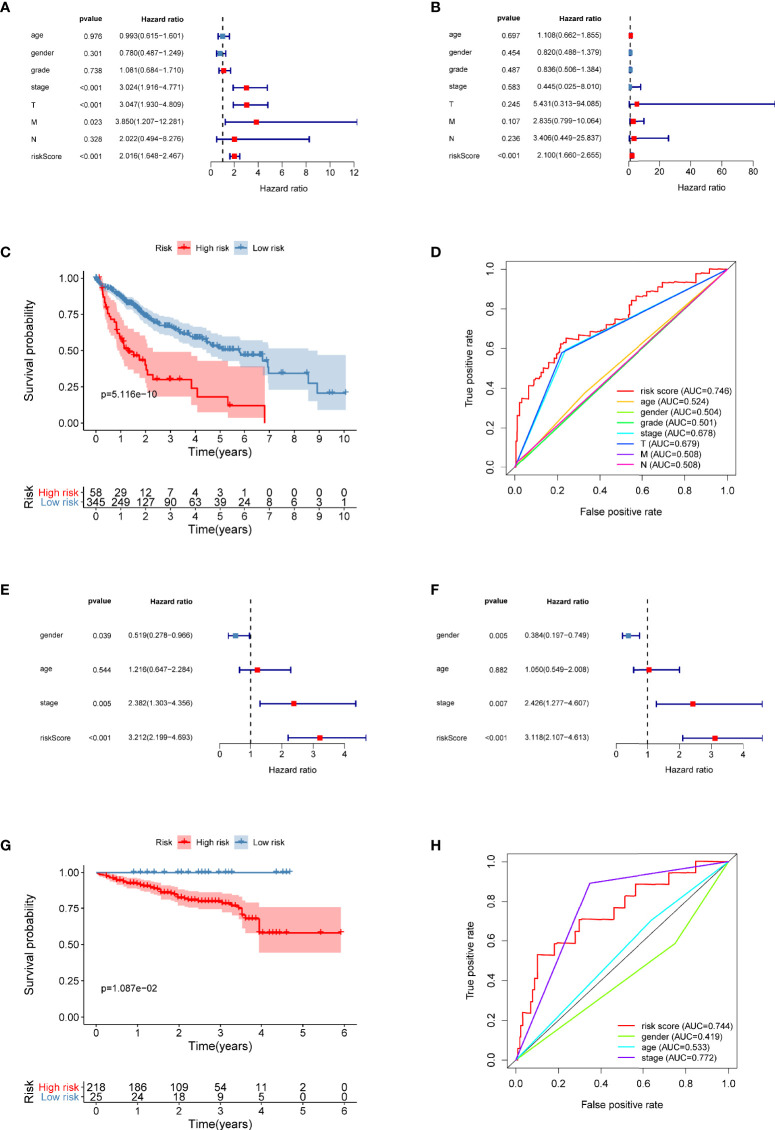
The prognostic value of the five-gene risk score for patients with liver cancer. **(A, B)** Univariate and multivariate Cox regression analyses revealed the independent value of the autophagy-related signature for overall survival (OS) of patients in TCGA database. **(C)** Kaplan–Meier (K–M) survival curve of OS of patients in the high-risk and low-risk patients based on TCGA database. **(D)** The time-dependent ROC curve analysis shows the AUC value for OS based on TCGA database. **(E, F)** Univariate and multivariate Cox regression analyses verified the independent value of the autophagy-related signature for OS of patients in the ICGC database. **(G)** K–M survival curve of OS in high-risk and low-risk patients based on the ICGC database. **(H)** Time-dependent ROC curve analysis shows the AUC value for OS based on the ICGC database.

Univariate and multivariate Cox regression models were utilized to compare clinical parameters and the independent predictive value of the autophagy-related risk score model. In TCGA dataset, the univariate Cox analysis revealed that the risk score, tumor stage, and T and M stages were correlated with the OS of patients with HCC (**Figure 8A**). The five-gene risk score remarkably correlated with survival in the multivariate Cox regression analysis, whereas other factors were not significant (**Figure 8B**). Both univariate and multivariate Cox regression analyses revealed that the risk characteristics, sex, and tumor stage of patients with liver cancer were markedly correlated with OS in the ICGC dataset (**Figures 8E, F**). These results confirmed that the autophagy-related prognostic model independently predicted the survival of patients with liver cancer.

The risk score of each patient in TCGA set was calculated with the five-gene risk score formula. As expected, the risk score classified patients with liver cancer into high- and low-risk groups based on the optimal cutoff point, and these groups had significantly different prognoses (**Figure 8C**). High-risk patients experienced a shorter OS than low-risk patients. The ICGC dataset was treated as an independent external validation set and was processed using the same method. The Kaplan–Meier analysis confirmed the prognostic ability of the signature once again (**Figure 8G**).

Using OS-based ROC curves, the predictive performance of the risk score was assessed. The AUC value for the risk score of OS in TCGA dataset was 0.746, which was apparently higher than that of age (AUC = 0.524), sex (AUC = 0.504), tumor grade (AUC = 0.501), tumor stage (AUC = 0.678), T stage (AUC = 0.679), M stage (AUC = 0.508), and N stage (AUC = 0.508) (**Figure 8D**). The AUC of the signature in the ICGC dataset was also as high as 0.744 (**Figure 8H**). Based on these data, the risk score was effective in predicting the survival of patients with liver cancer.

### FKBP1A Affects the Biological Behavior of Liver Cancer Cells *In Vitro* and *In Vivo*

**Figure 9:**
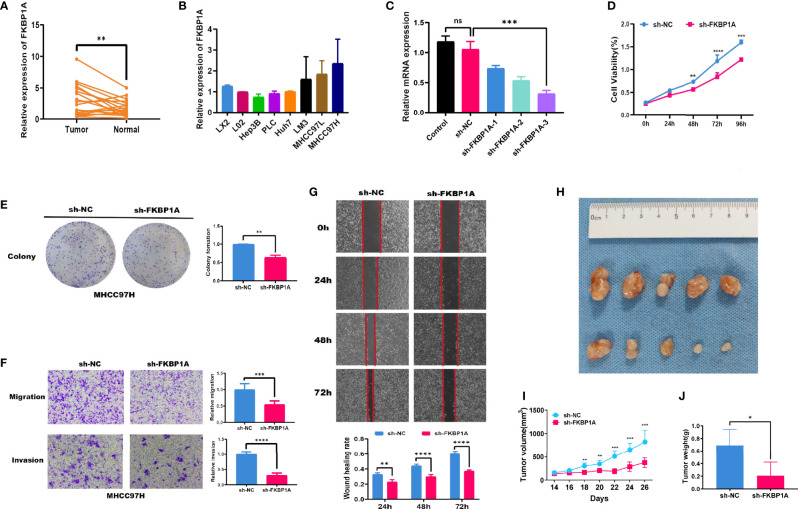
FKBP1A expression in HCC tissues and the effects of FKBP1A on HCC cell biological behaviors *in vitro* and *in vivo*. **(A)** qRT-PCR analysis of FKBP1A expression in HCC tissues from patients compared with adjacent non-cancerous tissues. **(B)** Levels of FKBP1A in HCC cell lines, a human hepatic stellate cell line, and a normal liver cell line. **(C)** qRT-PCR was performed to detect the efficiency of sh-FKBP1A-1, 2, and 3 transfection. **(D)** Growth curves for transfected MHCC97H cells were determined using the CCK-8 assay. **(E)** A colony formation assay was performed to assess the colony forming ability after transfection. **(F)** MHCC97H cells expressing a control shRNA or FKBP1A shRNA were subjected to Transwell migration and invasion assays. **(G)** Wound healing assays with MHCC97H cells expressing the control shRNA or FKBP1A shRNA. **(H)** sh-FKBP1A suppresses tumor growth *in vivo*. **(I)** The length and width of the tumors were measured using Vernier calipers. The tumor volume was calculated, and the tumor growth curves were plotted. **(J)** After 26 days, the nude mice were euthanized, and the tumor tissues were weighed. Ns, not significant, *p < 0.05, **p < 0.01, ***p < 0.001 and ****p < 0.0001. Data are presented as the means ± SD (n ≥ 3).

Twenty tumor samples and 20 samples of normal adjacent tissues from corresponding patients with HCC were tested using quantitative real-time PCR (qRT-PCR). FKBP1A and CAPNS1 were expressed at higher levels in tumors than in the normal tissues, but no significant differences in the expression of ATIC, BAX, and BIRC5 were observed (**Figure 9A** and **Figure S1**). The CPTAC database was used to analyze the expression of the FKBP1A protein in patients with liver cancer. In the matched samples, FKBP1A was expressed at higher levels in liver tumors than in adjacent tissues (**Figure S2**). In addition, the expression level of FKBP1A in six HCC cell lines (MHCC97H, MHCC97L, HuH7, LM3, Hep3B, and PLC) was compared with that in the human hepatic stellate cell line LX2 and normal human liver LO2 cell line using qRT-PCR. FKBP1A expression was significantly increased in MHCC97H cell lines compared with LX2 and L02 cell lines (**Figure 9B**). For the subsequent study, we selected MHCC97H cell lines transfected with shRNAs for FKBP1A gene knockdown experiments. The shFKBP1A-3 group was selected because the plasmid transfection efficiency was greater than 70% (**Figure 9C**). We performed proliferation, migration, and invasion experiments to assess the biological functions of FKBP1A in HCC cells. The CCK-8 assay performed to evaluate viability and cell proliferation and showed that FKBP1A knockdown prominently impaired MHCC97H cell growth (**Figure 9D**). Consistently, the results of colony formation assays showed that the number of cell clones decreased and clonogenic survival was inhibited following the downregulation of FKBP1A (**Figure 9E**). Transwell assays were performed to investigate the effects of FKBP1A on invasion and migration, which play important roles in cancer progression. The knockdown of FKBP1A expression impeded HCC cell migration and invasion compared with the sh-NC group (**Figure 9F**). Moreover, the migration of MHCC97H cells in the sh-NC and sh-FKBP1A groups was also assessed using a wound healing assay. FKBP1A knockdown significantly reduced the ability of cells to migrate (**Figure 9G**). Furthermore, we examined the role of sh-FKBP1A in the tumor growth of MHCC97H cells *in vivo*. As expected, the tumor size, tumor volume, and tumor weight in the sh-FKBP1A group were remarkably attenuated (**Figures 9H–J**). Therefore, sh-FKBP1A positively regulated malignant tumor behaviors *in vivo* and *in vitro*.

### FKBP1A Knockdown Increases the Autophagy Level in HCC Cells and Tissues

**Figure 10:**
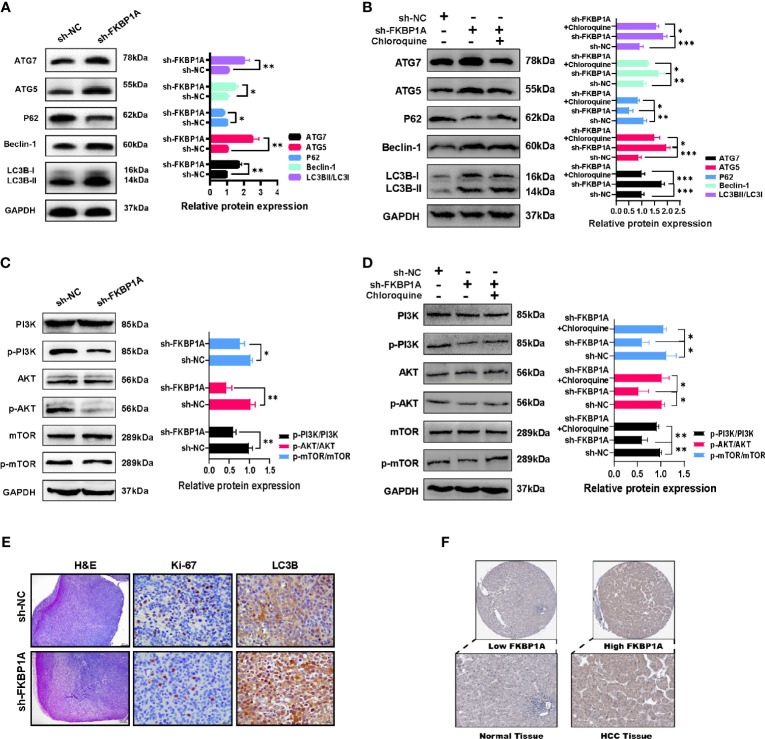
The inhibition of autophagy by sh-FKBP1A in HCC cell lines and mice. **(A, B)** Western blot analysis showing the levels of autophagy-related proteins in FKBP1A knockdown cells treated with or without chloroquine (10 µM). **(C, D)** Western blot results and analysis of mTOR, p-mTOR, PI3K, p-PI3K, AKT, and p-AKT levels in different groups treated with or without chloroquine (10 µM). **(E)** Histopathological changes were examined using H&E staining (bar = 200 μm). Expression levels of Ki-67 and LC3B in tumor tissues were detected using immunohistochemistry (bar = 20 μm). The presence of brown granules in the nucleus or cytoplasm was considered positive staining for target proteins. **(F)** IHC staining for FKBP1A in liver cancer and normal liver tissues. *p < 0.05, **p < 0.01 and ***p < 0.001. Data are presented as the means ± SD (n ≥ 3).

We investigated the levels of LC3B, p62, Beclin-1, ATG5, and ATG7 in MHCC97H cells to further discover the molecular mechanism underlying autophagy induced by decreased FKBP1A expression. Western blotting results showed that FKBP1A knockdown significantly increased the levels of the autophagic markers LC3B-II, Beclin-1, ATG5, and ATG7 and decreased SQSTM1/p62 levels (**Figure 10A**). Then, MHCC97H cells were pretreated with the autophagy inhibitor chloroquine, and the relevant proteins described above were analyzed using Western blotting. As shown in **Figure 10B**, chloroquine reversed the increases in the LC3B-II/LC3B-I ratio and Beclin-1, ATG5, and ATG7 levels induced by sh-FKBP1A. The level of p62 was decreased after treatment with sh-FKBP1A, which was also reversed by chloroquine. The PI3K/AKT/mTOR pathway is most closely related to autophagy. We assessed the levels of phosphorylated PI3K, AKT, and mTOR in HCC cells transfected with or without sh-FKBP1A to determine whether the FKBP1A-mediated regulation of autophagy might involve this signaling pathway. Our Western blot results showed reductions in the levels of p-PI3K/PI3K, p-AKT/AKT, and p-mTOR/mTOR in MHCC97H cells transfected with sh-FKBP1A (**Figure 10C**). Chloroquine was administered to the cells transfected with sh-FKBP1A to further explore the involvement of the PI3K/AKT/mTOR pathway in FKBP1A-mediated autophagy. The ratios of p-PI3K/PI3K, p-AKT/AKT, and p-mTOR/mTOR were consistently reduced in cells transfected with sh-FKBP1A, and these ratios were increased by combined treatment with chloroquine (**Figure 10D**), suggesting that FKBP1A-induced autophagy of HCC cells might or at least partially be mediated by the PI3K/AKT/mTOR pathway. In addition, the expression of Ki-67 and LC3B in HCC tumors from mice was assessed using IHC. We found that sh-FKBP1A inhibited the expression of Ki-67 and increased the expression of LC3B (**Figure 10E**). Furthermore, the HPA database was used to analyze immunohistochemical staining for FKBP1A. FKBP1A was expressed at higher levels in liver cancer than that in normal liver tissues (**Figure 10F**). Together, FKBP1A knockdown might block cell proliferation but induce autophagy by regulating the PI3K/AKT/mTOR signaling pathway.

## Discussion

HCC is one of the most lethal human cancers. With the development of clinical management strategies for liver cancer, some prognostic factors, including tumor volume, grade, and stage and the number of lesions, have been characterized (7, 8). However, an effective molecular biomarker for monitoring the HCC prognosis is still urgently needed. Based on accumulating emerging evidence, autophagy is closely related to tumorigenesis and progression (9). Explorations of the mechanism of autophagy provide new prospects for liver cancer treatment. Currently, high-throughput biological technologies have been widely applied in the early diagnosis of cancer. Therefore, using large-scale databases will help to explore the expression patterns of ARGs and reveal the prognosis of patients with HCC.

In the current study, based on high-throughput expression data from two datasets (TCGC and ICGC), we aimed to screen key ARGs that were strongly correlated with the prognosis of patients with HCC. First, 22 ARGs were differentially expressed between liver tumor and normal tissues, including 3 upregulated and 19 downregulated genes. The results of GO term and KEGG pathway analyses showed substantial enrichment in tumor biological processes and molecular functions. The results of the GO enrichment analysis suggested that 22 differentially expressed ARGs are related to phagocytosis and cell adhesion. Phagocytosis by macrophages plays an important role in the development of HCC. Inhibition of phagocytosis is associated with an increased risk of HCC growth and metastasis and correlates with shorter overall survival and recurrence-free survival in patients with HCC. Increased metastasis has been indicated to be associated with a poor prognosis for patients with HCC. Alteration of the cell adhesion system plays a central role in extrahepatic recurrence (4). The KEGG pathways in which the differentially expressed ARGs were enriched were identified in several types of cancer. Thus, a specific autophagy pattern may play a role in the occurrence and progression of liver cancer. Then, we observed 14 risk ARGs related to OS in TCGA database by dimensionality reduction in the univariate survival analysis. A subsequent multivariate survival analysis identified five key prognostic ARGs (ATIC, BAX, BIRC5, CAPNS1, and FKBP1A) that were used to construct prognostic risk models, which provided an accurate prognosis for patients with malignant liver tumors. Transcription factors that are closely related to these five genes were identified, and the results suggested that the transcription factor POU5F1 might be applied to predict the outcomes of patients with HCC. Multivariate Cox regression analysis of the prognostic risk model and clinical parameters showed that the five-gene risk score is conducive to independently predicting the prognosis of patients with liver cancer. Meanwhile, the results of the ROC curve analysis suggested that the prognostic risk model accurately distinguished healthy people from patients with liver cancer.

ATIC, a bifunctional protein enzyme, catalyzes the final two steps of the *de novo* purine biosynthetic pathway. According to recent studies, ATIC is expressed at high levels in lung cancer and is related to a poor patient prognosis (10). Few reports have described its function in liver cancer. The upregulation of ATIC in HCC is correlated with shorter survival and supports the propagation of HCC cells by regulating the AMPK-mTOR-S6 K1 signature (11).

BAX has been proven to be one of the most widely characterized proteins participating in autophagy. It is a member of the BCL2 protein family that functions as an apoptotic activator by forming a heterodimer with BCL2. Bax participates in mitochondria-initiated intrinsic apoptotic signaling and the extrinsic apoptotic pathway triggered by transmembrane death receptors (12, 13). Additionally, BAX affects the cross talk between the endoplasmic reticulum (ER) signaling pathway and mitochondrial pathways (14). Considering the important role for Bax in apoptosis, the finding that Bax regulates in the efficacy of multiple anticancer drugs that induce apoptosis of cancer cells is not surprising. SKA3, a component of the spindle and kinetochore-related complexes, influences cell apoptosis by regulating BAX/BCL-2 expression in HCC cells (15). The modulation of p53 expression by MT1G (a low-molecular-weight protein with high affinity for zinc ions) results in the upregulation of Bax, which leads to HCC cell apoptosis (16).

BIRC5 is a member of the inhibitor of apoptosis (IAP) family and prevents apoptotic cell death. BIRC5 exerts its effect on HCC cells by promoting proliferation (17). Recent studies have shown that BIRC5 should be regarded as a potential biomarker for molecular diagnosis and therapeutic intervention of HCC, and it exerts a significant effect on the prognosis of patients with HCC (18, 19).

CAPNS1, a member of the calpain small subunit family, operates as a heterodimer and is essential for calpain activity and function. Several studies have shown that CAPNS1 has biological functions in tumorigenesis. Indeed, CAPNS1 expression has been detected in various cancers, including hepatocellular carcinoma (20), ovarian carcinoma (21), colorectal cancer (22), and nasopharyngeal carcinoma (23). *In vitro* experiments revealed that CAPNS1 enhances the growth and metastasis of HCC by activating the FAK-Src signaling pathway and MMP2 (24).

FKBP1A is a cis-trans prolyl isomerase that binds to FK506 and rapamycin and inhibits calcineurin and mTOR activity (25). FKBP1A was validated to be overexpressed in HCC and predicted a poor prognosis (26). However, the specific mechanism of FKBP1A in HCC remains unclear and deserves further investigation.

At present, the specific molecular mechanism of FKBP1A in HCC is still not widely understood. Using TCGA and ICGC databases, we screened 5 differentially expressed ARGs, including FKBP1A, and established prognostic models of liver cancer. FKBP1A was expressed at high levels in HCC tissues and contributed to a poor prognosis. An analysis of related transcription factors revealed that POU5F1 may regulate CAPNS1 and FKBP1A expression, and high expression of POU5F1 is related to the poor prognosis of patients with HCC. POU5F1, a stemness-related transcription factor, was found to promote the liver cancer stem cell phenotype and cancer metastasis and regulate the expression of signature genes of HBV-derived HCC (27, 28). An analysis of the data in the CPTAC database showed that FKBP1A was also differentially expressed in HBV-related HCC. The key finding of the study is that FKBP1A expression was significantly upregulated not only in public databases but also in the HCC tissues we collected, indicating that FKBP1A may be a potential diagnostic marker. Consistently, FKBP1A was overexpressed in HCC cell lines, suggesting that FKBP1A may function as an oncogene in HCC development. *In vitro* cell function assays confirmed that FKBP1A knockdown significantly altered the biological behavior of liver cancer cells and that its downregulation decreased cell proliferation, migration, and invasion. Moreover, the loss of FKBP1A significantly inhibited HCC cell tumor growth in a nude mouse xenograft model. Our data also showed that low expression of FKBP1A induced autophagy *in vitro* and *in vivo via* the PI3K/AKT/mTOR pathway.

In summary, a close relationship exists between autophagy and the prognosis of patients with liver cancer. As shown in the present study, risk ARGs are potential candidate prognostic biomarkers for HCC with value in guiding decision-making regarding the choice of clinical treatment. At the same time, we validated the clinical and functional significance of FKBP1A, but further study is needed to elucidate a more detailed mechanism. However, the main limitation of our study is that we used available data from two public databases, and the results described above require further investigation in prospective studies.

## Data Availability Statement

The original contributions presented in the study are included in the article/**Supplementary Material**. Further inquiries can be directed to the corresponding authors.

## Ethics Statement

The studies involving human participants were reviewed and approved by the Ethics Committee in Clinical Research of the First Affiliated Hospital of Wenzhou Medical University. The patients/participants provided their written informed consent to participate in this study.

## Author Contributions

Conceptualization, WY. Software, ZZ. Validation, ZS, WY, and YLZ. Resources, QZ and BC. Data curation, ZS and YZ. Writing—original draft preparation, WY and ZS. Writing—review and editing, QZ. Supervision, QZ. All authors contributed to the article and approved the submitted version.

## Conflict of Interest

The authors declare that the research was conducted in the absence of any commercial or financial relationships that could be construed as a potential conflict of interest.

## Publisher’s Note

All claims expressed in this article are solely those of the authors and do not necessarily represent those of their affiliated organizations, or those of the publisher, the editors and the reviewers. Any product that may be evaluated in this article, or claim that may be made by its manufacturer, is not guaranteed or endorsed by the publisher.
