# Supplementary material for: Autophagy-Related Signatures as Prognostic Indicators for Hepatocellular Carcinoma
Source: Front Oncol. 2022 Mar 24;12:654449. doi: 10.3389/fonc.2022.654449 (PMC8987527; doi:10.3389/fonc.2022.654449)
Supplement: Supplementary file 1 [file DataSheet_1.docx]

**Table S1**. GO and KEGG analysis of differentially expressed autophagy-related genes

| **Category** | **ID** | **Term** | **P-value** | **Genes** |
| --- | --- | --- | --- | --- |
| Biological Process | GO:0018209 | peptidyl-serine modification | 1.43E-08 | BAK1, BAX, HSP90AB1, MAPK3, PARP1, PRKCD, RPTOR |
| Biological Process | GO:0043393 | regulation of protein binding | 1.87E-07 | BAK1, BAX, FKBP1A, HSP90AB1, MAPK3, PRKCD |
| Biological Process | GO:0051098 | regulation of binding | 1.91E-07 | BAK1, BAX, FKBP1A, HSP90AB1, MAPK3, PARP1, PRKCD |
| Biological Process | GO:0018105 | peptidyl-serine phosphorylation | 2.71E-07 | BAK1, BAX, HSP90AB1, MAPK3, PRKCD, RPTOR |
| Biological Process | GO:0006909 | phagocytosis | 1.46E-06 | CCL2, HSP90AB1, MAPK3, PRKCD, SPHK1, VAMP7 |
| Biological Process | GO:0007568 | aging | 1.75E-06 | BAK1, CANX, FOS, MAPK3, NAMPT, PRKCD |
| Biological Process | GO:0071496 | cellular response to external stimulus | 1.82E-06 | BAK1, FOS, ITGA6, MAPK3, NAMPT, RPTOR |
| Biological Process | GO:0007178 | transmembrane receptor protein serine, threonine kinase signaling pathway | 1.92E-06 | FKBP1A, FOS, HSP90AB1, ITGA3, MAPK3, PARP1 |
| Biological Process | GO:0070997 | neuron death | 2.91E-06 | BAX, CCL2, FOS, HSP90AB1, NAMPT, PARP1 |
| Biological Process | GO:0048284 | organelle fusion | 4.84E-06 | BAK1, BAX, SPHK1, VAMP7 |
| Cellular Component | GO:0031143 | pseudopodium | 0.000178 | MAPK3, VAMP7 |
| Cellular Component | GO:0046930 | pore complex | 0.0002 | BAK1, BAX |
| Cellular Component | GO:0008305 | integrin complex | 0.000565 | ITGA3, ITGA6 |
| Cellular Component | GO:0098636 | protein complex involved in cell adhesion | 0.000684 | ITGA3, ITGA6 |
| Cellular Component | GO:0098552 | side of membrane | 0.000746 | CANX, FKBP1A, ITGA3, ITGA6 |
| Cellular Component | GO:0005925 | focal adhesion | 0.001086 | CAPN1, ITGA3, ITGA6, MAPK3 |
| Cellular Component | GO:0005924 | cell-substrate adherens junction | 0.001106 | CAPN1, ITGA3, ITGA6, MAPK3 |
| Cellular Component | GO:0030055 | cell-substrate junction | 0.001158 | CAPN1, ITGA3, ITGA6, MAPK3 |
| Cellular Component | GO:0016323 | basolateral plasma membrane | 0.001626 | HSP90AB1, ITGA3, ITGA6 |
| Cellular Component | GO:0005912 | adherens junction | 0.002457 | CAPN1, ITGA3, ITGA6, MAPK3 |
| Molecular Function | GO:0051400 | BH domain binding | 7.18E-05 | BAK1, BAX |
| Molecular Function | GO:0046332 | SMAD binding | 0.000106 | FKBP1A, FOS, PARP1 |
| Molecular Function | GO:0051087 | chaperone binding | 0.000213 | BAK1, BAX, BIRC5 |
| Molecular Function | GO:0031072 | heat shock protein binding | 0.000354 | BAK1, BAX, HSP90AB1 |
| Molecular Function | GO:0070412 | R-SMAD binding | 0.000399 | FOS, PARP1 |
| Molecular Function | GO:0044325 | ion channel binding | 0.000436 | BAK1, FKBP1A, HSP90AB1 |
| Molecular Function | GO:0043236 | laminin binding | 0.000512 | ITGA3, ITGA6 |
| Molecular Function | GO:0050840 | extracellular matrix binding | 0.001403 | ITGA3, ITGA6 |
| Molecular Function | GO:0016763 | transferase activity, transferring pentosyl groups | 0.001536 | NAMPT, PARP1 |
| Molecular Function | GO:0050839 | cell adhesion molecule binding | 0.002326 | ATIC, HSP90AB1, ITGA3, ITGA6 |
| KEGG PATHWAY | hsa04210 | Apoptosis | 1.56E-08 | BAK1, BAX, BIRC5, CAPN1, FOS, MAPK3, PARP1 |
| KEGG PATHWAY | hsa05210 | Colorectal cancer | 1.36E-06 | BAK1, BAX, BIRC5, FOS, MAPK3 |
| KEGG PATHWAY | hsa05131 | Shigellosis | 1.26E-05 | BAX, CAPN1, CAPNS1, MAPK3, PRKCD, RPTOR |
| KEGG PATHWAY | hsa01524 | Platinum drug resistance | 2.29E-05 | BAK1, BAX, BIRC5, MAPK3 |
| KEGG PATHWAY | hsa04141 | Protein processing in endoplasmic reticulum | 3.44E-05 | BAK1, BAX, CANX, CAPN1, HSP90AB1 |
| KEGG PATHWAY | hsa04621 | NOD-like receptor signaling pathway | 5.21E-05 | CCL2, HSP90AB1, MAPK3, NAMPT, PRKCD |
| KEGG PATHWAY | hsa04215 | Apoptosis - multiple species | 5.50E-05 | BAK1, BAX, BIRC5 |
| KEGG PATHWAY | hsa05222 | Small cell lung cancer | 5.71E-05 | BAK1, BAX, ITGA3, ITGA6 |
| KEGG PATHWAY | hsa04657 | IL-17 signaling pathway | 6.21E-05 | CCL2, FOS, HSP90AB1, MAPK3 |
| KEGG PATHWAY | hsa04933 | AGE-RAGE signaling pathway in diabetic complications | 7.92E-05 | BAX, CCL2, MAPK3, PRKCD |
| KEGG PATHWAY | hsa05216 | Thyroid cancer | 8.56E-05 | BAK1, BAC, MAPK3 |
| KEGG PATHWAY | hsa04915 | Estrogen signaling pathway | 0.000276 | FOS, HSP90AB1, MAPK3, PRKCD |
| KEGG PATHWAY | hsa05213 | Endometrial cancer | 0.000329 | BAK1, BAX, MAPK3 |
| KEGG PATHWAY | hsa05224 | Breast cancer | 0.000351 | BAK1, BAX, FOS, MAPK3 |
| KEGG PATHWAY | hsa05223 | Non-small cell lung cancer | 0.000482 | BAK1, BAX, MAPK3 |
| KEGG PATHWAY | hsa04217 | Necroptosis | 0.000508 | BAX, BIRC5, HSP90AB1, PARP1 |
| KEGG PATHWAY | hsa05161 | Hepatitis B | 0.000508 | BAX, BIRC5, FOS, MAPK3 |
| KEGG PATHWAY | hsa05164 | Influenza A | 0.00061 | BAK1, BAX, CCL2, MAPK3 |
| KEGG PATHWAY | hsa05218 | Melanoma | 0.000623 | BAK1, BAX, MAPK3 |
| KEGG PATHWAY | hsa05214 | Glioma | 0.000702 | BAK1, BAX, MAPK3 |

| **Characteristics** | **Number (n = 20)** |
| --- | --- |
| Age |  |
| > 65 (n, %) | 5 (25.0%) |
| ≤ 65 (n, %) | 15 (75.0%) |
| Gender |  |
| Male (n, %) | 19 (95.0%) |
| Female (n, %) | 1 (5.0%) |
| Histological grade |  |
| G1 + G2 (n, %) | 18 (90.0%) |
| G3 + G4 (n, %) | 2 (10.0%) |
| Pathological stage |  |
| I + II (n, %) | 19 (95.0%) |
| III + IV (n, %) | 1 (5.0%) |
| T stage |  |
| T1 + T2 (n, %) | 12 (60.0%) |
| T3 + T4 (n, %) | 8 (40.0%) |
| N stage |  |
| N0 (n, %) | 20 (100.0%) |
| N1 (n, %) | 0 (0.0%) |
| M stage |  |
| M0 (n, %) | 20 (100.0%) |
| M1 (n, %) | 0 (0.0%) |

**Table S2.** Clinical characteristics of liver cancer patients.

| **Genes** | **Sequences** |
| --- | --- |
| ATIC | F: 5’-GACCTCATCGTAGCCACCATTGC-3’ |
|  | R: 5’-GCCGATAACCTGCCCGTTCTTG-3’ |
| BAX | F: 5’-GATGCGTCCACCAAGAAGCTGAG-3’ |
|  | R: 5’-CACGGCGGCAATCATCCTCTG-3’ |
| BIRC5 | F: 5’-TTCTGGGCTATGGGTGAGGTTCC-3’ |
|  | R: 5’-AGTTTGGCTTGCTGGTCTCTTCTG-3’ |
| CAPNS1 | F: 5’-GATAGCGACACCACAGGCAAGC-3’ |
|  | R: 5’-CAAAGGCACCTGGGAGTTCACTAC-3’ |
| FKBP1A | F: 5’-TGATCCGAGGCTGGGAAGAAGG-3’ |
|  | R: 5’-GAAGACGAGAGTGGCATGTGGTG-3’ |
| β-ACTIN | F: 5’-CCTGGCACCCAGCACAAT-3’ |
|  | R: 5’-GGGCCGGACTCGTCATAC-3’ |

**Table S3.** Primers sequences for qRT-PCR.

**Figure S1.** Expression level of ATIC (A), BAX (B), BIRC5 (C) and CAPNS1 (D) in normal tissues and cancers.

**
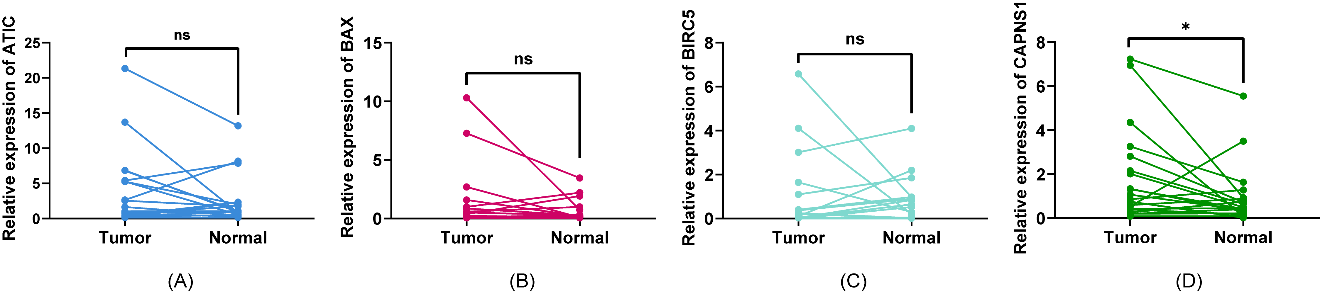
**

**Figure S2.** Expression level of FKBP1A in liver cancer and adjacent tissues.
